# Lying to an older adult in a sharing situation: differences between young and mid-life adults

**DOI:** 10.3389/fpsyg.2025.1541248

**Published:** 2025-06-18

**Authors:** Eitan Elaad, Yakir Bracha, Hodaya Avraham, Chen Rabi, Talya Katzin

**Affiliations:** Department of Psychology, Ariel University, Ariel, Israel

**Keywords:** lying, ageism, Ultimatum Game, age differences, Fake Fairness, Self-Reported Lying Scale

## Abstract

**Introduction:**

The present study aimed to demonstrate lying to older adults by young and mid-life participants in the Ultimatum Game (UG). Another goal was to reexamine the Self-Reported Lying Scale (SRLS), validate the short Hebrew version of the need for cognition scale (NCS-6), and show how they predict lying in the present experimental conditions.

**Methods:**

We allocated 379 examinees (196 women) to six experimental conditions in a 2 × 3 factorial design. Two participant’s age conditions (young and middle-aged) and three receiver’s age conditions (25, 50, and 70 years). Participants underwent a UG where they were permitted to conceal part of the endowment from the receiving woman. They then shared the remaining money with the receiver. Finally, participants completed the SRLS and the NCS-6.

**Results:**

Participants (mainly young) tended to evaluate an older woman less favorably than younger versions of that woman. Young participants concealed more of their endowment than mid-life participants. Young participants were more generous than their mid-life counterparts when sharing the remaining endowment with the older woman. Hiding a more significant part of the endowment while offering a fairer share of the remaining award (Fake Fairness) was observed for young participants. Fake Fairness to the older woman by younger participants was more significant than the receiver’s younger variations. The SRLS global score and four subscales predicted participants’ lying in the UG. NCS-6 prediction of lying was also significant, although less efficient than the SRLS.

**Discussion:**

The present study aimed to examine ageism by lying to an older woman in the UG. Indeed, young participants lied more to an older receiver than to younger versions of that receiver, whereas mid-life participants did not. We suggest that mid-life participants prepare themselves psychologically to join an older community and, therefore, are more tolerant toward older people than their younger counterparts. Young participants scored higher on the SRLS and lied more in the UG than mid-life participants. The present study contributes to a better understanding of the different approaches to lying by young and mid-life people. Young participants were relatively free to consider lying and behaving deceptively, whereas mid-life participants restricted their lying behavior and attitudes toward lying.

## Introduction

The present study aimed to examine how much money young and middle-aged participants would share with a woman who appeared young (25 years old), mid-aged (50 years old), or older (70 years old).

The question of how younger people see and treat older people is important because, in Western societies, the proportion of people defined as old (65 + years) proliferates. Likewise, ageism, thinking or believing negatively about becoming old or about older adults and avoiding being old, is growing, too. Modernization or technological progress may explain the stereotypical attitude against people in late adulthood. Younger people are more adjusted to rapid technological changes than older people, which dictates a decline in the status of the older. Raynor (2015) added that older adults have difficulty finding gainful employment. People see them as technology-averse, unwilling to learn new skills, challenging to manage, too expensive, and needing to be more productive to justify the perceived increased expense.

Furthermore, modern demands prefer youthful alertness over mature experience (Nelson, 2005), and the increased proportion of older unemployed people results from positive stereotypes of younger age, such as creativity, superior learning of new skills, and quick decision-making (Abrams et al., 2016). Chopik and Giasson (2017) found that younger adults expressed more explicit attitudes against the old, whereas older adults showed a more implicit bias. Schüttengruber et al. (2022) reported negative attitudes toward older people (80 and older) among Austrian students.

Bratt et al. (2020) referred to studies that compared modern societies in evaluating older adults (North and Fiske, 2015; Vauclair et al., 2015) and indicated that cultural individualism in these societies developed increased tolerance and respect for older people. Bratt et al. (2020) contended that the increased structural support for older people generates positive (rather than adverse) attitudes toward the older. Verissimo et al. (2022) found variability in age-related changes across attention/executive functions. Some decline with age, while others improve. For example, they noted that older adults showed better orienting attention and ignoring distractions than middle-aged adults.

The belief that older people have a degraded lie-detection ability and, therefore, would not retaliate when being lied to is another aspect of ageism. Consequently, older people are often the victims of financial abuse by people in a position of trust, such as relatives and caregivers (McGreevey, 2005; Tueth, 2000) or by con artists who tend to exploit them financially (Caslo et al., 2020; James et al., 2014; Sweeney and Ceci, 2014). For example, Morgan and Tapp (2024) referred to an account saying that in 2020, there were 105,301 fraud cases against older people over 60 in the US. Also, older people’s belief in fake news contributed to the reduced lie-detection ability attributed to them (Guess et al., 2019).

Studies have examined the validity of the belief about reduced lie-detection ability in older people with conflicting results. On the one hand, Bond et al. (2005) reported that older females were more accurate at detecting deception in young senders than older males and younger participants of either sex, implying that the ability to spot lies improves with age due to experience. Shaw and Lyons (2017) supported the idea that deception detection accuracy increases with age.

In contrast, O'Connor et al. (2019) showed that older participants (age 66–89) who viewed truthful and deceptive child interview videos were less accurate in their credibility evaluations than young participants (age 18–30). Older participants showed more substantial truth bias and greater confidence than younger adults. A meta-analysis on trust by Bailey and Leon (2019) showed that older adults were more trusting than young adults.

Stanley and Blanchard-Fields (2008) reported that older participants performed worse on lie detection than younger participants due to reduced emotion recognition related to poor visual capacity. Ruffman et al. (2012) compared older (aged 60 to 89 years) and younger (aged 17 to 26 years) judges on detecting deception and concluded that the older participants were worse at detecting rehearsed (not spontaneous) lies. Sweeney and Ceci (2014) showed that college students (ages 18–23) were better lie detectors of spontaneous pro-social lies than older adults (ages 60–93). Furthermore, the older the adult was, the worse the ability to detect lies. The results suggest that the ability to detect lies may decrease with age due to cognitive decline.

Slessor et al. (2014) noted that own-age biases are involved in detecting deception. Specifically, older people are likely to trust those of their age and trust younger speakers less. Older participants also showed more confidence in their judgments of their age members than other-age speakers. The researchers did not observe similar own-age biases for younger participants.

Ruffman et al. (2008) noted that older people were worse than their younger counterparts in recognizing emotions such as anger, sadness, fear, or happiness in body expressions and in recognizing anger in vocal expressions. Murphy and Isaacowitz (2010) explained that older people have difficulties identifying emotional expressions that may help detect inconsistencies between the content of the message and the body language. Due to physical changes, older adults are susceptible to deception (Castle et al., 2012).

Caslo et al. (2020) suggested that the human brain’s prefrontal cortex supports detecting deception, which changes with age. They showed that the ability to detect deception declined after age 65 and even more after age 80.

Contrary to the prevailing belief, Dimelow (2018) found that the ability to recognize emotion in older adults (aged 59 to 84) can be maintained and even improved. Dimelow offers hope, suggesting that older adults’ lie-detection ability remains intact.

Chen et al. (2023) indicated that people are likelier to deceive those older than them, whereas older people trust people younger than them. They explained that with age, the increased trust in others may stem from older people’s higher motivation to derive emotional meaning and reduce information processing that signals risk (Mata et al., 2016).

Finally, Horta et al. (2024) showed that younger adults performed better than older adults on trust-related decision-making. More research is necessary on the association between ageism and lying to older people, and we accepted the challenge.

### Middle adulthood

Following Slessor et al. (2014), we assumed that an own-age bias exists among mid-life but not younger participants by which mid-life participants would favor their own-age receiver. To examine the bias, we included mid-life participants in the study.

Middle adulthood (or mid-life) is the lifespan between young and old, commonly defined as 40 to 65 years. The mid-life person has settled down, is economically active, and expects to live longer as part of an older community.

Mid-life is the least studied period and much remains to be done for future research. As we do not know much about the lying behavior of this age group compared to other ages, we conducted this study to examine mid-life adults’ lying behavior to younger, own-age, and older receivers.

### Sharing resources in the UG

The original form of the UG (Güth et al., 1982) granted one player (the sender) some monetary endowment to share with a partner (the receiver). Not knowing the receiver’s identity, senders were free to share the endowment. The receiver can accept or reject the sender’s offer but cannot suggest a different proposal. The two players obtain their agreed share if the receiver accepts the offer. In case of rejection, both players receive nothing.

Since then, substantial research examined multistage bargaining (Thaler, 1988), and the interplay between fairness and reason. For example, Novak et al. (2000) showed that fairness will evolve if the sender receives information about the responder’s acceptance of past deals.

Relevant to the present study is the asymmetric information version of the UG (e.g., Elaad et al., 2024; Vesely, 2014), which provides the opportunity to adhere to honest sharing or to conceal part of the endowment for the participant’s benefit.

The asymmetric version investigates deception by keeping the sender’s endowment amount private. The sender can declare any amount of endowment to the receiver and keep the concealed part for themselves. Then, the sender makes an offer to the receiver, who must decide whether to take or reject it. If the receiver turns the offer down, both players go empty-handed. Results indicated that, on average, senders claim to have received a lower endowment than they indeed had.

The present study generated three age looks of the same woman through a free picture adopted from the internet, which was manipulated by free AI programming to change the ages of that woman. We provided participants with the manipulated picture and a written description of the woman’s age while granting them a sum of 100 NIS (about $30). We asked participants to offer all or part of the endowment for sharing with the receiver woman. Specifically, they could conceal a portion of the endowment from the receiver and keep it for themselves.

We examined whether an older woman receiver would trigger more significant lies (concealment) about the monetary endowment than younger versions of that woman.

Note that the experimental rules allowed deception, and the participant did not experience exposure concerns.

Finally, group moderators such as religiosity might influence our receiver conditions. Precisely, Jewish religious participants who endorse respect for the elders would lie less to the older version of the woman than their secular counterparts.

### Fake Fairness and lying to older people in the UG

Fake Fairness in the UG is an interplay of the concealment magnitude and the allegedly fair sharing of the remaining endowment. Participants eager to conceal the most and then showed extreme kindness in offering the remaining award are assigned the highest Fake Fairness score, which reflects another lying type.

Elaad et al. (2024) noted that it is essential to study Fake Fairness because it is a widespread practice online where low stakes, permission to lie, and anonymity prevail. They referred to Drouin et al. (2016), who examined online deception across four different online sites (i.e., social media, online dating, anonymous chat rooms, and sexual websites). Drouin et al. (2016) showed that most of their participants reported lying online, and a large majority suspected that others lied too online. They concluded that people lie because everyone lies on the internet. We expect that people will Fake Fairness online. For example, retailers offer a deal with a significant discount (equivalent to fair sharing) while not including substantial product or service components, for which the recipients would be charged extra.

However, anonymity and permission to lie are not preconditions to Fake Fairness. Service providers may charge an initial low wage (pretending to be fair), thus buying the recipient’s trust and controlling the successive interaction that benefits the service provider by mediating between the recipient and retailers. For example, a construction supervisor charges a fair price for supervising the construction of a building, being aware that the owner will need building materials, and the supervisor will benefit by recommending the dealer and receiving a commission from the dealer.

Fake Fairness is fundamental when considering the belief about reduced lie-detection ability in older people. Lie detection failure is consistent with the belief that older people would not retaliate when deceived by others. As a result, older people are often victims of financial abuse. Ebner et al. (2020) studied spear-phishing online attacks and suggested age effects on susceptibility to online deception. They showed higher susceptibility in older people and attributed it to lower short-term episodic memory.

Nevertheless, there are different views about the validity of the idea that older people are less equipped than younger people to detect lies. Besides studies that imply a deterioration in the lie-detection ability with age, which is explained by a cognitive decline (Ruffman et al., 2012; Stanley and Blanchard-Fields, 2008), other accounts indicated that older people improve their lie-detection ability due to experience (Bond et al., 2005; Shaw and Lyons., 2017).

The cognitive decline explanation for older people’s vulnerability to lying is unclear. Difficulties in identifying emotional expressions that typify older people (Murphy and Isaacowitz, 2010) support the cognitive decline explanation. The difficulties stem from changes in the human brain’s prefrontal cortex that develop with age (Castle et al., 2012). However, a different view shows that the ability to recognize emotion can be preserved and sometimes improve with age (Dimelow, 2018).

### The Self-Reported Lying Scale

The Self-Reported Lying Scale (SRLS) presents five features relating to lies. Unlike other questionnaires that seek to measure the respondent’s dispositions, the SRLS allows responders to present themselves better than they are and support their self-image.

Elaad et al. (2024) introduced the SRLS and reported that the SRLS global score and some subscales predicted the extent of the concealed endowment. The predictions highlight various aspects of lying behavior. To reexamine these predictions, we included the scale in the current study.

Elaad et al. (2024) described the five sub-scales in length. Hereafter, we will highlight some of the main characteristics of each scale.

One of the five SRLS’s lying features is the self-assessed ability to tell lies convincingly. The feature is subjective and does not necessarily reflect successful lying behavior. Usually, people assign average and even low ratings to their lying abilities (see Elaad, 2018a for a review), which serves to maintain their honest self-image. The self-assessed lie-telling ability correlated positively with narcissistic features (Zvi and Elaad, 2018) and negatively with religiosity (Elaad, 2018b). More importantly, higher rankings of the lie-telling ability are associated with reports of actual lying (Zvi and Elaad, 2018; Verigin et al., 2019). Examples of the ability to tell lies convincingly are “My friends believe me when I lie” and “I find it easy to convince others with my lies.”

A second feature of the SRLS is the subjective self-assessed ability to detect lies efficiently. People tend to assess this ability higher than the scale midpoint (see Elaad, 2018a for a review). Recently, Fernandes et al. (2023) observed that almost 80% of their participants indicated that they could detect lies, reporting an average success score close to 70%, well above the chance level of 50%. Nevertheless, Bond and DePaulo (2006) found that people’s lie detection performance is around the chance level, which is also true for professionals (Burgoon et al., 2021). Relying on wrong cues (Aavik et al., 2006), combining many, sometimes conflicting, cues into the veracity judgment (Street and Richardson, 2015), and using weak cues (Verschuere et al., 2023) may explain this failure. Examples of the lie detection ability statements are: “People agree that I am an able lie-detector” and “I find it easy to uncover other people’s lies.”

The third SRLS feature is applying rationality when lying. The feature is rated above the scale’s midpoint three and predicted enhanced lying in the UG (Elaad et al., 2024). The results highlighted individual differences in rational processing while lying, corresponding with the broader notion of individual differences in rational processing (Cacioppo et al., 1996). Examples of rationality in lying statements are “I try to be rational when I lie” and “My lies are thoughtful.”

Another SRLS attribute corresponds to people’s attitude toward the acceptability of deception. McCornack and Levine (1990) demonstrated individual differences in finding deception acceptable. Oliveira and Levine (2008) showed that lie acceptability is positively related to narcissism and negatively associated with religiosity. Quinn et al. (2023) associated lie-acceptability with Machiavellianism and functional impairment at work, home, and social settings. However, ratings might be biased since people rate their ethical behavior higher than they should (Tenbrunsel et al., 2010). Examples of lie-acceptability statements are “It is OK to lie to achieve your goals” and “There is nothing wrong with not telling the truth now and then.”

Finally, the SRLS uses self-assessed frequent lying. Gneezy et al. (2013) identified variations in individual lying over time in economic interactions. Zvi and Elaad (2018) found that reports of frequent lying correlated with narcissism.

The mean score of frequent lying is lower than the SRLS midpoint 3 (Elaad et al., 2024), indicating that people are reluctant to report frequent lying to protect their honest self-view. Examples of frequent lying statements are “I have no problem telling many lies” and “People say I lie a lot.”

We expect people who use small lies to embellish their self-presentation as good lie tellers, suitable lie detectors, and rational liars to gain more money in the UG. We also expect those who approve of lying and admit to lying frequently to lie more in the UG than lower scorers.

### Need for cognition

The Need for Cognition is a personality feature reflecting the extent to which individuals pursue and enjoy thinking and the effort they invest in cognitive activities. Cacioppo and Petty (1982) generated the Need for Cognition Scale (NCS), which consisted of 34 questions to measure the feature. Later, Cacioppo et al. (1984) shortened the scale to 18 items. Higher NCS scores are associated with an increased appreciation of debate and problem-solving. Lower scorers process information more heuristically, with little elaboration. Finally, the NCS is free of gender influences.

Studies used the 18 items of the NCS to examine how jurors’ need for cognition affects their legal decisions (Bornstein, 2004) and how their need for cognition influences the self-reports of students’ satisfaction with life (Coutinho and Woolery, 2004). However, the results of Reinhard (2010) are particularly pertinent to the present study. Reinhardt’s study found that the NCS was a strong predictor of success in lie detection, with higher NCS scorers demonstrating more success than lower NCS scorers in classifying truthful and deceptive messages.

More recently, Coelho et al. (2020) introduced a shorter version of the NCS with only six statements and called it the NCS-6. They noted that while the NCS-6 saves time, the cost in construct validity with variables such as openness, cognitive reflection test, and need for affection is minimal. They found solid psychometric evidence for using the NCS-6 across the US and the United Kingdom and concluded that the NCS-6 is a reliable and valid measure of the need for cognition.

Usually, lying is cognitively more demanding than telling the truth (Deeb et al., 2020), and liars may start thinking about creating an impression of credibility. Therefore, the NCS-6 may help predict the lying magnitude in the UG.

The following is a summary of our hypotheses:

1. Participants would conceal more money from an older woman than from younger appearances of that woman.
2. Compared to mid-life participants, younger participants would conceal more money from the endowment and, particularly, deny more resources from the older woman. Following Slessor et al. (2014), we expect mid-life participants to conceal less from their age than from another age receiver.
3. We expect participants to Fake Fairness, defined as the tendency to offer a fairer partition of the remaining endowment as concealment grows.
4. We will find that the SRLS and NCS-6 scores have predictive power in the UG, particularly about lying behavior.

## Methods

### Design

We assigned participants to six experimental conditions in a 2 × 3 between-subject factorial design, with two participants’ age conditions (young and mid-life) and three receivers’ age conditions (25, 50, and 70 years). The allocation of participants to the receivers’ age conditions was random.

### Statistical power and participants

We examined 379 Jewish Israeli participants (196 females) recruited from the local community through the media and a snowball method. The sample consisted of 170 secular and 209 religious participants. We employed two age groups: 199 young participants aged 20–35 (mean age 25.2, SD 3.36) and 178 mid-life participants aged 40–61 (mean age 50.4, SD = 6.69). We excluded two participants whose ages did not fit in our groups. All participants volunteered to participate in the study and gave their informed consent. We determined the sample size following Elaad et al. (2024), who looked for a medium effect size of 0.30 to detect UG effects. Using a G*Power analysis for correlation and regression tests (Faul et al., 2009) showed that a sample of 111 participants would be appropriate for a study that considers power (1-*β*) > 0.95, *α* = 0.05, and *f* = 0.30. Our two main factors (two age groups of participants and three ages of the receiver) satisfied these demands.

## Materials

### The Self-Reported Lying Scale

Participants completed the Self-Reported Lying Scale (SRLS) (Elaad et al., 2024). The scale consists of 20 statements, and we asked participants to evaluate to what extent they agree or disagree with each. Participants responded on a 5-point Likert scale ranging from 1 (strongly disagree) to 5 (strongly agree), with intermediate levels of 2 (disagree), 3 (no opinion), and 4 (agree).

### NCS-6

NCS-6 comprises six statements (Coelho et al., 2020). Participants answered on a 5-point Likert scale (1 = extremely uncharacteristic of me; 5 = extremely characteristic of me). Examples of NCS-6 statements*: I would prefer complex to simple problems,* and *I like to have the responsibility of handling situation that require a lot of thinking.* We translated the short NCS-6 into Hebrew, and the present study provides an opportunity to look at its reliability and validity as a lying predictor.

### Positive and negative trait questionnaire

We asked participants to rate the receiver woman on a list of six positive and six negative traits: Dubious, Friendly, Reliable, Tensed, Pleasant, Restricted, Happy, Inviting, Sad, Lonely, Receptive, and Detached. We asked participants how indicative each trait is. Participants responded on a 5-point Likert scale ranging from 1 (not at all) to 5 (very much) with the following intermediate levels: 2 (slightly), 3 (no opinion), 4 (indicative).

### Procedure

The ethics committee of Ariel University approved the study. Through social networks, we recruited participants from the broader Israeli community and told them that the study was about sharing. After agreeing to participate, an experimenter contacted the participants and met them in person (85%) or in a Zoom meeting (15%). The two meeting types were approximately the same across age groups. We used *Google Forms* to deliver the experiment and present the photos. We asked participants to sign a consent form indicating that their identity would be kept secret and that they could end their participation in the study at any time without penalty. We presented participants with a brief background questionnaire (gender, age, religiosity). We then randomly divided the sample into three parts, with an equal number of men and women in each part. We presented them with a UG where they shared money (100 NIS, about $35 at the time of the study) with a woman receiver who appeared in three age variations (Supplementary Figure 1). We performed the meetings under close surveillance of the experimenter. Following the UG, we asked participants to rate the receiver on six positive and six negative traits. Finally, we asked participants to complete the SRLS and NCS-6.

The game procedure is analogous to Elaad et al.'s (2020). As such, the description of the procedure partly overlaps. Each participant received the following instructions: “In this experiment, your partner is a 25-year-old woman whose photo is enclosed (in other conditions, the age changed to 50 or 70). A sum of 100 NIS is allocated to both of you. Only you know this is the sum of money to be shared. The other woman does not know the starting sum of money and is unaware of a fair division. You should propose the sum of money to give her, bearing in mind that the deal will be completed if only she accepts your offer. Your aim is to keep as much money as possible for yourself. To this end, you may inform your partner that the sum of money to be shared is less than 100 NIS. If she accepts your offer, you will receive the agreed money. In addition, you will receive the money you concealed from her. If she rejects your offer, neither of you will receive any money, and both will be declared losers. Remember that she cannot suggest a different money division and can only accept or reject your offer. To ensure you understand the rules, please answer the following two questions before continuing:

Assume that the sum of money you have decided to share with that woman is 80 NIS.

If you offer 30 NIS to her and she accepts your offer, you receive.

_____________ NIS, and she receives _____________ NIS.

If you offer 30 NIS to her and she rejects your offer, you receive.

_____________ NIS, and she receives____________ NIS.

Now, you must decide what to offer your partner. Below, enter the best offer you believe will likely be accepted by her.

The number of allocated NIS for sharing is _____________.

I keep ________________ NIS for myself and offer her ___________ NIS. The final two numbers should equal the sum of allocated money.

After completing the task, we thanked and debriefed the participants.

We informed participants that the endowment reflected actual money, and three participants winning a drawing would receive the funds to which they were entitled. Indeed, after completing the study, we performed a drawing with a random number program to decide the three winners and pay them.

## Results

### Evaluating the receiver

We asked participants to evaluate the receiving woman on 12 personal traits: six positive features: friendly, reliable, pleasant, happy, inviting, and receptive, and six negative ones: dubious, tensed, restricted, sad, lonely, and detached. Participants made their evaluations on a five-point Likert scale. We computed each participant’s average positive and negative evaluations and then summarized them across participants. To generate a unified measure, we reversed the negative evaluations by subtracting them from 6 and added the resulting scores to the positive evaluations. High scores indicate positive evaluations. Table 1 presents the means of the unified scores obtained for young and mid-life participants and each age description of the receiver woman.

**Table 1:** Means (and SD) of the evaluations made by younger and mid-life participants for three receiver’s age conditions.

Receiver’s age	25	50	70	Across
Young participants (20–35)				
Mean (SD)	3.52 (0.56)	3.59 (0.62)	3.32 (0.61)	3.47 (0.60)
N	71	62	66	199
Mid-life participants (40–61)				
Mean (SD)	3.66 (0.63)	3.83 (0.66)	3.61 (0.59)	3.70 (0.63)
N	60	60	57	177
Across participants				
Mean (SD)	3.59 (0.59)	3.71 (0.65)	3.45 (0.62)	(0.63) 3.58
N	131	122	123	376

Larger scores stand for more positive evaluations.

We conducted a 2 × 3 between-subject ANOVA on the mean evaluation scores. The two factors are the participant’s age (young and mid-life participants) and the three receiver age variations (25, 50, and 70) of the same woman. A significant receiver’s age effect emerged, *F*_(2,370)_ = 5.02, *p* = 0.007, η_p_^2^ = 0.026, indicating differences in the evaluations of the three receiver’s age versions. Next, we applied a planned Helmert contrast to the receiver’s age appearance. The first contrast compared the evaluation of the older woman (70) with the evaluations of the two other age groups. The contrast revealed that the evaluation of the older woman was significantly less positive (contrast estimate = −0.189, *p* = 0.005). Comparing the evaluations of the 50-and 25-year-old receivers provided no significant difference. The participant’s age effect was also significant, *F*_(1,370)_ = 4.64, *p* < 0.001, η_p_^2^ = 0.033, indicating that mid-life adults were more positive than younger adults in evaluating the receiving woman (Table 1).

Inspection of Table 1 shows the considerable contribution of the young participants to less-positive evaluations. Separating the two age groups of participants and contrasting the receiver age evaluations show that young participants evaluated the 70 years woman less positively than the other two age descriptions (contrast estimate = −0.234, *p* = 0.01). Comparing the 25-and 50-year-old receivers revealed no significant difference. We applied similar contrasts to the mid-life participants and found no significant difference between the older woman’s evaluation and the other two age descriptions (contrast estimate = −0.130, *p* = 0.20). However, Table 1 reveals that mid-life participants evaluated the 50-year-old woman more favorable that either the young or the older woman which corresponds to our second hypothesis. We contrasted the evaluation of the 50-year-old receiver with the evaluations of the 25-and 70-year-old receivers and obtained a significant difference (contrast estimate = 0.204, *p* = 0.043).

To conclude, people (especially young people) tend to consider an older woman less favorably than the younger appearances of this woman. Mid-life participants tend to evaluate the own-age woman more favorably than the younger and older receivers. The question is, would the attitudes reinforce lying to the less favorable age of the receiver with a focus on the older woman?

### Concealing money in the UG

The concealed sum of money in the UG may provide an answer. We describe the level of concealment (lying) in Table 2.

**Table 2:** Means (and SDs) of the concealed sum of money in the UG computed for younger and mid-life participants and three receiver age variation.

Receiver’s age	25	50	70	Across
Young participants (20–35)				
Mean (SD)	25.38 (27.1)	22.87 (27.4)	32.26 (30.6)	26.88 (28.5)
N	71	62	66	199
Mid-life participants (40–61)				
Mean (SD)	12.17 (21.2)	13.67 (20.8)	10.52 (19.4)	12.13 (20.4)
N	60	60	58	178
Across participants				
Mean (SD)	19.33 (25.3)	18.32 (24.7)	22.09 (28.1)	3.58 (0.63)
N	131	122	124	377

We conducted a 2 × 3 between-subject ANOVA (two levels of the participant’s age and three levels of the appeared receiver’s age) with planned Helmert contrasts for the receiver’s age conditions. We added two levels of religiosity (secular and religious) entered as a covariate. A significant participant’s age effect emerged, F _(1,370)_ = 34.42, *p* < 0.001, η_p_^2^ = 0.085, revealing that mid-life participants concealed less money from the receiving woman than younger participants (Table 2). Religiosity also showed a significant effect, F _(1,370)_ = 4.59, *p* = 0.033, η_p_^2^ = 0.012. Although small, the effect indicates that religious participants concealed less from the receiving woman (mean = 17.7, SD = 25.1, *N* = 209) than secular participants (mean = 22.6, SD = 26.9, *N* = 170). A closer look at the three-receiver age conditions and the age of the participants reveals the contribution of the 50-year-old receiver condition to mid-life participants’ secular-religious difference (mean = 22.8, SD = 25.2, *N* = 29, and mean = 5.2, SD = 10.3, *N* = 31, respectively). The difference is significant (t _(58)_ = 3.58, *p* < 0.001, *d* = 0.93). All other receiver age conditions and participant’s age groups generated no significant secular-religious differences. The considerable effect size may imply that mid-age secular participants concealed more money from the 50-year-old woman than mid-age religious participants who are relatively sensitive to receivers of the same age (Slessor et al., 2014). However, when we compared the 50-year-old condition with the other two receiver conditions, secular mid-age participants showed a Helmert contrast estimate of −9.67 and *p* = 0.062. It is unclear why mid-life secular participants prevented more resources from a receiver similar to their age than from other receivers.

We conducted a similar analysis with gender (men and women participants) and obtained no significant difference in the concealed amount of money. Across religiosity and gender, the woman’s age showed no significant effect on concealment. Finally, we obtained no significant interaction effects. Table 2 shows that the young participants tended to conceal more money from the older woman. We applied a separate one-way ANOVA with planned contrasts on the receiver’s age conditions for each participant’s age group. The mid-life group showed no significant effects. The younger participants showed a Helmert contrast estimate of −8.14 and *p* = 0.059 when we compared the older woman and her younger appearances. The outcome did not reach significance by a slim margin.

### Fair sharing

We defined *Fair Sharing* as the money the participants offered the receiving woman divided by the money of the revealed endowment they kept for themselves. The larger the proportion, the fairer the sharing, as participants retain a smaller portion of the money. Statistics of fair sharing appear in Table 3.

**Table 3:** Means (and SDs) of fair sharing in the UG computed for younger and mid-life participants and three receiver’s age variations.

Receiver’s age	25	50	70	Across
Young participants (20–35)				
Mean (SD)	0.92 (0.33)	0.81 (0.27)	1.03 (0.40)	0.92 (0.35)
N	71	62	66	199
Mid-life participants (40–61)				
Mean (SD)	0.91 (0.20)	0.91 (0.26)	0.86 (0.20)	0.89 (0.22)
N	60	60	58	178
Across participants				
Mean (SD)	0.92 (0.28)	0.86 (0.27)	0.95 (0.33)	0.90 (0.30)
N	131	122	124	377

The higher the score, the fairer the sharing.

We performed a 2 × 3 ANOVA on the fair sharing means with two between-subject factors: the participant’s age (younger and mid-life participants) and the age variation of the receiving woman (25, 50, 70). We obtained a significant interaction effect, *F*_(2,158)_ = 6.27, *p* = 0.002, η_p_^2^ = 0.033. Table 3 shows enhanced fairness to the older woman by younger participants, whereas mid-life participants were less fair toward the older woman. We separated the younger and mid-life participants and applied planned contrasts on fair sharing with the receiver. Considering the young participants, comparing the older woman and her younger versions elicited a significant difference (Helmert contrast estimate = 0.158, *p* = 0.003). Results indicated that younger participants were significantly fairer with the old than the younger versions of that woman. We contrasted the 25- and 50-year-old versions; no significant difference emerged. We found no significant contrasts for the mid-life participants.

Inspecting Tables 2, 3 reveals that fair sharing mirrors the participant’s tendency to conceal more money from the receiver. The correlation between the concealed sum of money and fair sharing of the remaining endowment is r = 0.314, *p* < 0.001. Specifically, participants who concealed a more significant portion of the endowment would display a more favorable offer of the remaining endowment to secure it from rejection (Ding et al., 2014). In blunt words, the generous distribution of resources hides deception. Following Elaad et al. (2024), we call the phenomenon Fake Fairness.

### Fake fair sharing

Following an earlier Fake Fairness manifestation (Elaad et al., 2024), we merged significant concealment and staged fair sharing into a unified measure called Fake Fairness. First, we computed standard scores relative to the respective means and standard deviations across all participants for concealment and fair sharing. Second, we added the two standard scores to generate a combined score and ensure that each factor weighed equally in the new index. In that way, high Fake Fairness scores reflect a joint event of considerable concealment and a staged fair sharing of the remaining endowment.

We performed a 2 × 3 ANOVA on Fake Fairness means (Table 4), with two levels of participant’s age and three levels of receiver’s age variations. A significant participant’s age effect emerged, *F*_(1,371)_ = 16.24, *p* < 0.001, η_p_^2^ = 0.042, indicating that mid-life participants showed less Fake Fairness than younger ones. A significant interaction effect, F _(1,371)_ = 5.94, *p* = 0.003, η_p_^2^ = 0.031, suggests that young participants directed their highest Fake Fairness toward the older woman. In contrast, mid-life participants show their lowest Fake Fairness toward that woman (Table 4). We found no significant receiver age effect.

**Table 4:** Means (and SDs) of Fake Fairness Z-scores computed for younger and mid-life participants and three receiver age variation.

Receiver’s age	25	50	70	Across
Young participants (20–35)				
Mean (SD)	0.25 (1.76)	–0.20 (1.65)	0.86 (2.16)	0.31 (1.91)
N	71	62	66	199
Mid-life participants (40–61)				
Mean (SD)	–0.30 (1.15)	–0.22 (1.13)	–0.52 (1.10)	–0.35 (1.13)
N	60	60	58	178
Across participants				
Mean (SD)	–0.00 (1.53)	–0.21 (1.41)	0.21 (1.88)	–0.00 (1.62)
N	131	122	124	377

Higher positive scores reflect higher levels of Fake Fairness.

The interaction effect justifies separating between younger and mid-life participants. We conducted a separate one-way ANOVA (receiver’s age) on Fake Fairness with Helmert planned contrasts. Results indicated a significant receiver age effect for young participants, *F*_(2,196)_ = 5.25, *p* = 0.006, η_p_^2^ = 0.051. The difference is significant when we compared the Fake Fairness directed at the older woman with that of the two younger appearances of that woman (contrast estimate = 0.840, *p* = 0.003). Specifically, young participants show more Fake Fairness to the older woman than to her younger variations. We found no significant difference between the 25-and the 50-year-old receivers. The analysis conducted for the mid-life participants resulted in no significant outcomes.

### SRLS

Another goal of the present study was to reexamine the SRLS and use the results to assist us in understanding and explaining the lying behavior in the UG. To this end, we computed the mean and SD for each participant across the 20 statements (individual global score). Then, we recorded the means and SD of the individual scores across participants. We performed a similar procedure for each subscale; Table 5 presents the results. We included a 95% confidence interval in the table based on standard error units, which we computed for the total and the five subscale scores. Finally, we generated the reliability of the SRLS statements using the Cronbach alpha procedure. We displayed the reliability scores in Table 5.

**Table 5:** Statistics for the SRLS total score and five subscale scores.

	N of items	Mean (SD)	95% CI	Cronbach’s α
Global score	20	2.81 (0.61)	2.75–2.88	0.88
Tell lies	4	3.12 (0.96)	3.03–3.22	0.83
Detect lies	4	3.19 (0.87)	3.10–3.28	0.81
Rationality	4	3.77 (0.83)	3.68–3.85	0.69
Lie acceptability	4	2.17 (0.88)	2.08–2.26	0.79
Frequent lying	4	1.80 (0.80)	1.72–1.88	0.78

*N* = 375.

Table 5 shows good Cronbach’s alpha reliability for the global score and subscales. The exception is the rationality scale, which presented lower reliability. Elaad et al. (2024) reported similar lower reliability for that scale.

As to the averages, the ability to tell lies was within the midpoint range of 3 (as the confidence interval presents a lower bound smaller than three and an upper bound larger than 3). The results agree with Elaad et al. (2024) and earlier findings suggesting that people assign average ratings to their ability to tell lies convincingly. Following Elaad et al. (2024), the self-assessed lie-detection ability and the rating of being rational when lying generated higher average scores than the midpoint (the lower bound of the confidence interval is larger than 3). Analogous to Elaad et al. (2024), we expected lie acceptability ratings and frequent lying scores to be lower than the scale midpoint, which occurred (the upper bound of the confidence interval is smaller than 3).

Following Elaad et al. (2024), we correlated the five subscales of the SRLS and found that all 10 correlations were positive. Using Bonferroni correction for alpha inflation, nine correlations were significant. The exception was the correlation between the lie-detection ability and reported frequent lying (the recorded significance level was *p* = 0.06).

We then examined the SRLS prediction of the amount of concealed money in the UG. To this end, we applied linear regression analyses for the global score and its five subscales. Specifically, we entered the global score and every individual subscale as independent variables. We display the results in Table 6.

**Table 6:** Linear regression statistics describing SRLS predictions for concealing money from the receiver in the UG.

	*R* ^2^	β	t	Sig.
Global score	7.8	0.279	5.62	< 0.001
Tell lies	2.4	0.154	3.02	0.003
Detect lies	0.0	0.032	0.62	Ns
Rationality	4.8	0.219	4.34	< 0.001
Lie-acceptability	10.0	0.317	6.46	< 0.001
Frequent lying	7.1	0.266	5.34	< 0.001

*N* = 375.

After employing the Bonferroni correction, results indicated that the SRLS global score and four subscales significantly predicted participants’ lies in the UG. The attributed lie detection ability is irrelevant to the present task and does not predict lying.

### NCS-6

We first computed the scale statistics across participants. The mean score was 3.56 (SD = 0.87), with a 95% confidence interval of 3.65–3.47. The statistics indicate that participants accredited themselves with a high need for cognition (the lower bound of the confidence interval is larger than the scale midpoint 3). Finally, we computed Cronbach’s alpha reliability score, which was 0.85.

We correlated the NCS-6 score with the rational SRLS subscale score and found a significant correlation, r _(374)_ = 0.226, *p* < 0.001. We expected the outcome since the rational subscale has much in common with the need for cognition. The NCS-6 correlated with the global SRLS score, r _(375)_ = 0.164, *p* < 0.001, with the ability to tell lies, r _(374)_ = 0.172, *p* < 0.001, and the ability to detect lies, r _(374)_ = 0.178, *p* < 0.001.

We hypothesized that the NCS-6 scale would predict concealing in the UG. To examine the hypothesis, we applied a linear regression analysis in which we entered the mean NCS-6 scores as the independent predictor of lying. Results show that although the prediction is significant, *β* = 0.135, t = 2.63, *p* = 0.009, it explains only 1.8% of the variance in the participant’s lying. Results suggest the association between higher NCS-6 scores and concealing more money in the UG.

### SRLS differences in predicting lying

Following the ability of the scales to predict lying, it is worthwhile to examine possible differences in the scale’s prediction in the three-receiver age conditions. To this end, we conducted separate regression analyses for each of the three age appearances of the woman. Table 7 presents the relevant statistics for the global SRLS and the NCS-6 scores.

**Table 7:** Linear regression statistics computed for the global SRLS and NCS-6 predictions about concealing money in three-receiver age conditions.

	*R* ^2^	*β*	*t*	Sig.
SRLS Global score 25	2.7	0.164	1.88	0.063
SRLS Global score 50	8.3	0.288	3.29	0.001
SRLS Global score 70	5.2	0.229	2.57	0.011
NCS-6 25	1.0	−0.099	−1.12	0.263
NCS-6 50	2.7	0.163	1.81	0.073
NCS-6 70	4.2	0.204	2.28	0.025

The Bonferroni correction sets significance level at 0.017.

Table 7 shows that after applying the Bonferroni correction, the SRLS global score predicts lying to 50-and 70-year-olds but not to the young woman. The Need for Cognition Scale score did not predict significant lying.

The lie-telling ability assessment and the self-assessed lie detection ability failed to predict lying in any age condition, and therefore, we did not include them in Table 8.

**Table 8:** Linear regression statistics for predictions about concealing money in three-receiver age conditions by rationality, lie acceptability, and frequent lying.

	*R* ^2^	*β*	t	Sig.
Rationality 25	3.4	0.185	2.13	0.035
Rationality 50	5.9	0.243	2.74	0.007
Rationality 70	3.5	0.186	2.08	0.040
Lie acceptability 25	2.1	0.144	1.64	0.103
Lie acceptability 50	10.7	0.327	3.80	< 0.001
Lie acceptability 70	4.6	0.214	2.40	0.018
Frequent lying 25	0.1	0.069	0.78	0.435
Frequent lying 50	2.2	0.147	1.63	0.105
Frequent lying 70	6.5	0.256	2.90	0.004

The Bonferroni correction sets significance level at 0.017.

Applying the Bonferroni correction shows that being rational while lying and lie acceptability predicts lying to the 50-year-old receiver. The self-reported frequent lying subscale predicted lying only when the older woman was the receiver.

Finally, Table 9 presents participants’ age differences in scoring the SRLS, and we also compared secular and religious participants to generate a 2 × 2 between-subject factorial design for each SRLS subscale. We observed that secular participants scored consistently higher on the SRLS subscales than their religious counterparts. After applying the Bonferroni correction, the difference is significant for lie telling, *F*_(1,370)_ = 11.92, *p* < 0.001, η_p_^2^ = 0.031, lie detection, F _(1,370)_ = 10.58, *p* < 0.001, η_p_^2^ = 0.028, rationality, F _(1,370)_ = 19.7, *p* < 0.001, η_p_^2^ = 0.049, lie-acceptability, F _(1,370)_ = 40.36, *p* < 0.001, η_p_^2^ = 0.098, and the global score, *F*_(1,372)_ = 37.51, *p* < 0.001, η_p_^2^ = 0.092. Across religiosity, younger participants tended to score higher on SRLS subscales than mid-life participants. The exception is the lie detection ability subscale. The Bonferroni correction leaves us with a significant difference for lie frequency, F _(1,370)_ = 9.31, *p* = 0.002, η_p_^2^ = 0.025. The global score is also significant, F _(1,370)_ = 6.40, *p* = 0.012, η_p_^2^ = 0.017. We obtained a significant participant’s age and religiousness interaction effect for the rationality subscale, F _(1,370)_ = 7.30, *p* = 0.007, η_p_^2^ = 0.019, due to higher scoring of religious young participants than mid-life religious participants, t (_205_) = 3.55, *p* < 0.001, *d* = 0.50, and for the global score F _(1,372)_ = 6.77, *p* = 0.01, η_p_^2^ = 0.018. Whereas no difference exists between secular younger and mid-life participants’ scoring, religious young participants scored globally higher than mid-life participants, t (_205_) = 4.10, *p* < 0.001, *d* = 0.58. We found no other significant interaction effects.

**Table 9:** Means (and SD) of the SRLS subscale scores computed for younger and mid-life secular and religious participants.

Participants	Telling lies	Detecting lies	Rationality	Lie acceptability	Frequent lying	Global score
Young						
Secular Mean (SD) *N* = 83	3.28 (0.98)	3.27 (0.90)	3.96 (0.69)	2.55 (0.90)	2.00 (0.89)	3.01 (0.62)
Religious Mean (SD) *N* = 116	3.11 (0.97)	3.10 (0.87)	3.82 (0.78)	2.11 (0.84)	1.86 (0.85)	2.80 (0.58)
Across Mean (SD) *N* = 199	3.18 (0.97)	3.17 (0.88)	3.88 (0.74)	2.29 (0.89)	1.92 (0.87)	2.89 (0.61)
Mid-life						
Secular Mean (SD) *N* = 84	3.34 (0.87)	3.43 (0.87)	3.98 (0.70)	2.38 (0.88)	1.91 (0.74)	3.01 (0.57)
Religious Mean (SD) *N* = 91	2.84 (0.96)	3.01 (0.86)	3.39 (0.94)	1.71 (0.69)	1.46 (0.56)	2.48 (0.51)
Across Mean (SD) *N* = 175	3.08 (0.95)	3.21 (0.87)	3.67 (0.88)	2.03 (0.85)	1.68 (0.69)	2.73 (0.60)
Across age						
Secular Mean (SD) *N* = 167	3.31 (0.92)	3.35 (0.86)	3.97 (0.69)	2.46 (0.89)	1.96 (0.82)	3.01 (0.60)
Religious Mean (SD) *N* = 207	2.99 (0.97)	3.06 (0.87)	3.63 (0.88)	1.94 (0.80)	1.69 (0.76)	2.66 (0.57)

## Discussion

We investigated possible age differences in lying to a receiver in the UG. We found that participants, particularly young ones, translate their negative attitude toward older people into practice, such as lying to an older receiver more than to the younger looks of that receiver. Specifically, participants favored the older woman less than the younger looks of that woman, and referring to Fake Fairness, they lied more to the older receiver than the younger ones.

Fake Fairness denotes the link between concealing more from the receiver and boosting fair sharing of the remaining endowment. In generating Fake Fairness, we presented Ding et al.'s (2014) contention that fear of rejection, rather than concern for fair sharing, is a significant driver of the offering behavior. Ding et al. (2014) reported that Machiavellian personality traits are associated with fake honest sharing behavior. Nevertheless, the fear of rejection does not match the premise that we expect the older receiver to retaliate less than the younger ones.

An alternative explanation associates Fake Fairness with feelings of guilt. Specifically, concealing a large portion of the endowment from the receiver raises feelings of guilt, and participants are inclined to compensate for the guilt by a fairer sharing of the remaining award. This explanation better describes why participants who concealed more from the older woman would reduce guilt by offering a more significant portion of the remaining endowment. Future research should resolve which explanation better describes Fake Fairness in the UG, emphasizing the belief that older people have a reduced lie detection ability and, therefore, would retaliate less when someone lies to them.

### Mid-life and younger participants

We hypothesized that younger participants would conceal more money from the endowment than mid-life participants. Results supported the hypothesis.

We obtained equivalent results when we considered Fake Fairness. We found that mid-life participants showed less Fake Fairness than young ones and that young participants focused their Fake Fairness on the older receiver. Although not significant, mid-life participants showed the least Fake Fairness to the older receiver.

The results are associated with participants’ rating of the receiver’s dispositions in three age forms. Mid-life adults showed more positive attitudes toward the older woman than younger participants. Furthermore, young participants favored the 70-year-old woman less than the other two receiver ages, whereas mid-life participants showed no significant difference.

Following Slessor et al. (2014), we expected mid-life participants to conceal less from an own-age receiver than from receivers in the other two age conditions. Supporting this line of reasoning, we found a tendency among mid-life participants to evaluate the 50-year-old woman more favorably than that woman’s younger and older presentations. However, they showed no reduced rate of lying to the own-age receiver compared to the other-age conditions.

Differences in SRLS scores may partly explain the differences in lying between young and mid-life participants. The consistently higher SRLS scores by young compared to mid-life participants imply that young participants feel relatively free to consider lying (lie acceptability), hesitate less to report their lying behavior (lie frequency), and apply reason when lying. Mid-life participants are more restricted in this matter. Experience and wisdom may account for the relatively controlled attitude of the mid-life participants. Another speculative explanation is that people in middle adulthood prepare themselves psychologically to become part of an older community in the future, which dictates a more lenient attitude toward older people.

The present study contributes to understanding mid-life adults’ attitudes toward lying compared to the younger group. Other than studying lying, more research is necessary on many aspects of mid-life people’s attitudes and behavior.

### Religiosity

Finally, we assumed that group moderators such as religiosity might influence lying to the older receiver. Specifically, Jewish religious participants educated to respect older people would lie less to the older version of the receiver than secular participants. Results showed that religious participants concealed less than their secular counterparts, but we observed no religiosity differences in allocating money to the older receiver. We may explain the results by religious people’s limited cognitive flexibility, which the Jewish religious rules block. Cognitive flexibility is the ability to restructure knowledge in multiple ways with the demands of the changing situation (Spiro et al., 1995). We find support for cognitive flexibility differences in the NCS-6’s first statement, “I would prefer complex to simple problems.” Complex problems demand cognitive flexibility, whereas simple problems do not. Indeed, secular participants outperformed religious participants in their rating (mean = 3.44, SD = 1.17 and mean = 3.10, SD = 1.16, respectively). The difference is significant, *F*_(1,375)_ = 8.18, *p* = 0.004, η_p_^2^ = 0.02. Since lying is denounced by Jewish religious rules, religious participants tend to follow them without questions and lie less.

### SRLS and NCS-6

SRLS statistics replicated those in an earlier account (Elaad et al., 2024). The mean scores computed for all five subscales were similar. The similar Cronbach alpha reliability scores and the significantly positive intercorrelations of the five subscales contributed to the reliability of the SRLS.

We compared the scoring of the SRLS of young and mid-life participants and found that young participants scored higher on the SRLS than mid-life participants. The differences emerged when considering rationality, lie acceptability, and lie frequency reports, the same three subscales that predicted lying. We obtained no significant participant age differences for self-assessed abilities to tell and detect lies, which failed to predict lying. We looked further into differences in religiosity on SRLS scoring and found a significant difference in four subscales between secular (higher) and religious participants’ scoring (lower). Religious rules treat lying negatively, and therefore, religious participants ascribe themselves less lying in the various features of the SRLS than their secular counterparts. However, young religious participants are careful not to admit using spontaneous lies. When they must lie, they make it deliberately and thoughtfully in the limited conditions where and when lying is allowed.

The SRLS provides measures for a subjective view of lying. We did not expect responses to convey the actual attitudes of the responder but rather the attitudes that responders allow themselves to release concerning each statement. Responders hesitate less when addressing statements about applying rationality when lying, as demonstrated by the higher average score than the scale midpoint. They have difficulties disclosing that they accept lying and that they lie frequently. We observed these difficulties in scores lower than the scale’s midpoint.

Considering the receiver age variations, the SRLS subscale of rationality predicted lying to the 50-year-old receiver, whereas the Bonferroni correction denied the significance of lying to the other two age groups. Nevertheless, we may associate the results with the ease with which people admit to being rational when lying. Participants with fewer barriers against lying have less hesitation in admitting to being a rational liar when asked. When it comes to actual actions, they lie more. Participants with difficulties about lying would be less inclined to admit being rational when committing an act with which they disagree. When they face an opportunity to benefit from lying, they will hesitate and probably not lie or lie less.

People have difficulties accepting lies and, more so, admitting that this is their view. Only a fraction of people admit that they approve of lying. This fraction lied more to the 50-year-old receiver. It is unclear why they lie less to the other two groups of receivers.

Not many people admit that they lie frequently while probably preserving an honest self-image (Mazar et al., 2008). These few people are inclined to lie more to the woman whom they do not expect to retaliate and reject their offer, namely the older woman. They lie less to the young versions of that woman because they fear rejection.

We added a short version of the need for cognition scale (NCS-6). Higher scores are associated with more appreciation of the cognitive effort applied in information processing, whereas lower scorers process information more heuristically, with little elaboration. We suggested that liars in the UG should start thinking about creating an impression of credibility (i.e., Fake Fairness), which is cognitively demanding. Therefore, the NCS-6 may help predict the lying magnitude in the UG.

The NCS-6 mean scores showed that participants assigned themselves a high need for cognition score, and the reliability of the six scale’s items was satisfactory. We correlated the NCS-6 score with various SRLS scores and obtained significant relationships. The correlation with the rational SRLS subscale stands out since both have much in common.

We observed no significant associations between higher NCS-6 scores and lying to the different age appearances of the receiver.

### Limitations and suggestions for future research

The present study deviated from the usual UG procedure that brings together two real people. Instead, we presented our receiver with a photo, which may have affected the money sharing. The direction of influence is unclear yet, leaving room for additional research to clarify it.

Note that we administered the SRLS after the UG. Therefore, the scale’s predictions may not be independent of the money sharing.

Other group differences, in addition to age, may influence resource sharing with older adults. For example, religious participants lied less to the older receiver than secular ones. Similarly, we advise future research to look for differences in education, social status, and culture.

The sex dyad may also influence the decision to lie in the UG. We used a woman as a receiver in the present study. Therefore, our results and conclusions about lying to an older woman should be limited to women receivers. Lying to an older man may change the results and should be left to future research.

We should have used a third group of older participants to complete the present study design. Technical difficulties prevented us from doing it. We advise future researchers to compare a group of older participants with mid-life and young participants.

Cultural factors may limit the external validity of the present results. We used an Israeli sample with unique characteristics to refer to a woman at different ages. Research in various cultures is necessary to establish a broader view of lying to older people. Furthermore, we presented the SRLS and NCS-6 in Hebrew to Hebrew-speaking participants. More research in different languages is necessary to validate the current scale’s prediction of lying in the UG.

## Data Availability

The raw data supporting the conclusions of this article will be made available by the authors, without undue reservation.
