# Supplementary figures and images for: Lying to an older adult in a sharing situation: differences between young and mid-life adults
Source: Front Psychol. 2025 Jun 18;16:1541248. doi: 10.3389/fpsyg.2025.1541248 (PMC12213739; doi:10.3389/fpsyg.2025.1541248)

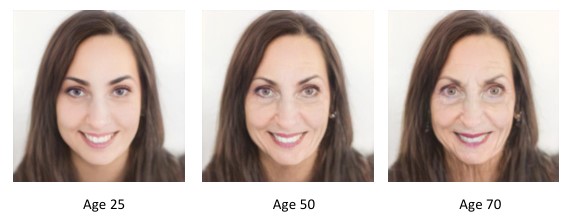

Supplement: Supplementary file 1 [file Image_1.jpeg]
